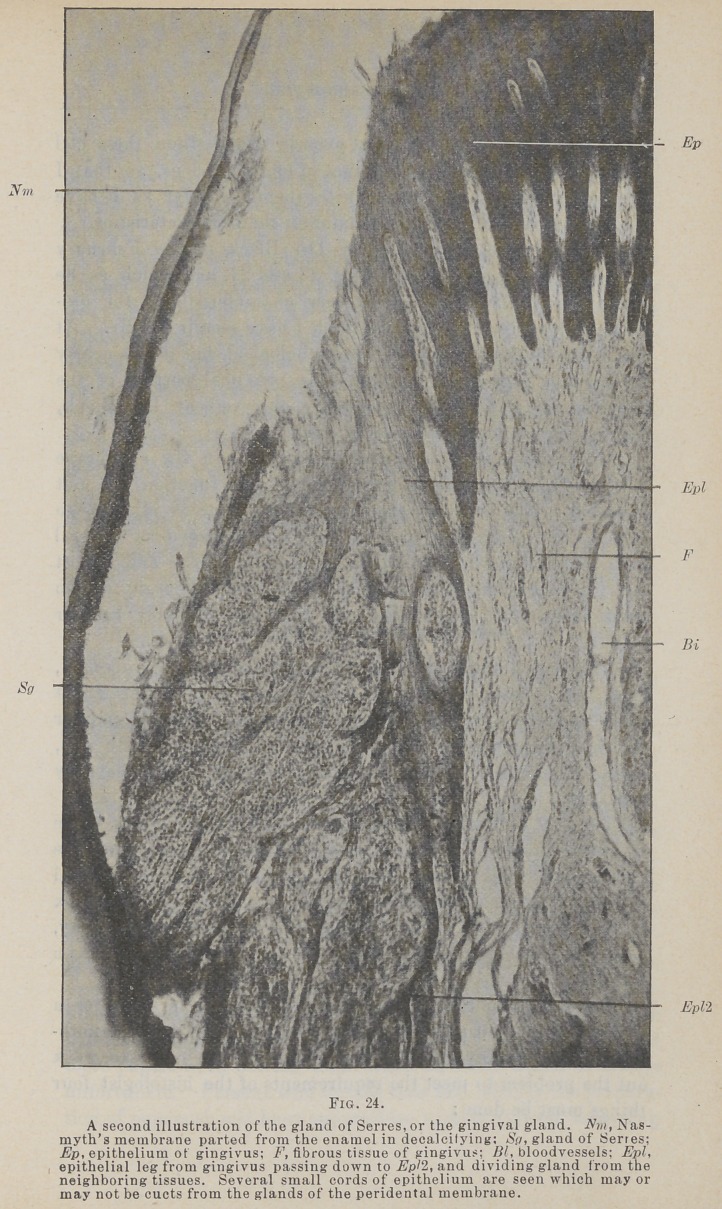# Epithelial Structures in the Peridental Membrane

**Published:** 1899-08

**Authors:** Frederick B. Noyes

**Affiliations:** Chicago. Ills.


					﻿Epithelial Structures in the Peridental Membrane.
BY FREDERICK B. NOYES, D.D.S., CHICAGO, ILLS.
Abstract of paper read before the Section of Stomatology, American Medical
Association, Columbus, June, 1899.
For four or five years I have been interested in the histologi-
cal study of the peridental membrane, and during that time have
devoted what time I could to the special study of certain struct-
ures found in that membrane, and called by Dr. Black, who
first described them, the glands of the peridental membrane.
The work which I have tried to do is really not in shape to
report, as it has not been worked out to the point where positive
statements can be made in regard to the nature of these struct-
ures.
I have decided to make this simply a report of my work and
a statement of the problem as it stands. The histological study
of this tissue is beset with the greatest technical difficulties.
One man has said to me that he had never seen a specimen of
tooth that would be considered technically acceptable in the
study of the liver, for instance.
It is almost impossible to get sections of the peridental mem-
brane as thin as would be desirable for high-power work.
Though they arc harder to study, many things can be learned
from thick sections; some things better than from thin ones,
especially by comparison with lower powers and the use of the
binocular. The difficulty of showing in photographic illustra-
tions the things that are learned in this way is very great,
however.
The diseases of the peridental membrane have attracted a
great deal of attention, and provoked an immense amount of
writing and discussion. It is impossible that anything satisfac-
tory can be worked out in regard to these conditions, which are
of so great interest to the dentist, until the problems are attacked
in a more scientific and rational manner. Until then we are
fighting we know not what, we know not how.
The Fig. 1 and 2 longitudinal section, Fig. 3 transverse
section peridental membrane, may be defined as the tissue which
fills the space between the root of the tooth and the bony wall
of its alveolus, being attached to the cementum on one side and
the bone on the other. It surrounds and is attached to the root
from the border of the alveolus to the gingival line, and supports
the epithelium of the gingivus. It has been called by a number
of names, of which I prefer pericementum, or peridental mem-
brane, the two being used synonomously. It belongs to the class
of fibrous membranes, being composed chiefly of white, fibrous,
connective tissue. It is not in any sense a double membrane,
and, while it has qualities in common with the periosteum, with
which it blends at the rim of the alveolus, it differs markedly
from the periosteum in any position.
In transverse sections of the membrane, which have been
well stained with haematoxylin and eosin, even with as low a
power as a thirty-five m. m. lens, small, deep stained bodies can
be seen lying close to the cementum, and winding between the
fibers as they spring from it. With a one-half or three-fourths
inch (Fig. 5) objective and a binocular instrument, the winding
of these cords of deep stained cells among the fibers is beautifully
shown. In such observation these bodies suggest very strongly
such structures as the sweat glands. As many as 200 bits of
these cords have been counted in a transverse section of the gin-
gival portion of the membrane around an incisor of a young
lamb.
In studying the arrangement of these cords they are found to
form a net-work about the root of the tooth, extending from
near the attachment of the epithelium at the gingival line almost
to the apex. In the gingival portion they form a close, meshed
net which grows more open as they pass apically. In sections
cut tangentially to the root, Fig. 6, this branching and net for-
mation is shown, but the entire arrangement can not be shown
in photograph. This diagram, Fig. 7, made by Dr. Black some
time ago, shows the plan as it is made out from the study of
many sections.
When Dr. Black first described these structures, thirteen or
fourteen years ago in his “ Studies of the Periosteum and Peri-
dental Membrane,” he considered them to be of lymphatic char-
acter, and there are things about them that support this idea
yet; but from a close study of the character of the cells they
appear to be of epithelial order, they show various forms, some-
times appearing ovoid, but usually polyhedral or cuboidal. The
neucleus is always large and conspicuous and often shows neu-
cleoli, Figs. 8, 9.
The cells are not arranged into true tubules in all places,
Fig. 13, though what appears to be a lumen, with a circle of
cells about it, may be found in a good many positions. The
structures are better described as cords of cells than as true,
distinct tubules.
The cords of cells lie very close to the cementum, rig. 16,
between the fibers as they spring from it, swinging out from the
root and back again in loops. In many places the end next to
the cementuni is club shaped, Figs. 17, 18, and come very close
to the root between the cementoblasts.
A delicate basement membrane surrounds these cords, Fig.
17, and in a few places a circular arrangement of fibers may be
seen about the large ones, Fig. 20.
I have searched for something in the form of a duct for these
structures, or some connection between the epithelium lining,
the gingival space and the cords. Some appearances which
suggest a duct are uniformly found, but it has been impossible
to follow them because of the failure in obtaining complete series
of sections.
In the gingival portion of the membrane, in transverse sec-
tions, I have found a number of very perfect tubuIeB in sections,
of which Fig. 20 is the best illustration I have been able to get,
but that is not as good a representation of the object as I could
wish. With the microscope it shows a perfect circle of cuboidal
cells with large nuclei. In the lumen are several loose cells.
There is a distinct basement membrane and a few circular fibers..
Just on one side of this is a small duct made up of four cells.
When these tubules have been observed, they show a tendency
to swing out from the surface of the root. The epithelium of
the gingivus presents long, slender projections, often of compli-
cated form. The connective tissue between these contains small,
round cells. This collection of round cells is especially conspicu-
ous on the proximal sides, and constitutes what has been called
the gingival gland.
The ducts have been followed among these epithelial legs,
where they have been lost. But even in this position their cell
structure is very different from that of epithelial legs, so that I
would say they do not connect with them. As far as I have
been able to follow them, they maintain their characteristics.
The structure referred to by Dr. Black in the February
Cosmos as the gingival gland, Figs. 23 and 24, and which as he
states is not a gland at all, it is very characteristic of the gin-
givas, at least in the sheep, in which I have seen it chiefly. At
first I was inclined to regard it as pathological, but it is so nearly
universal in large or small form on the proximal portion of the
gingivus, and has been so universally observed by Dr. Black,
that it seems to be the normal condition.
The presence of these epithelial structures in the membrane
is beyond question. Their nature, origin and function ca>; not
be stated. I have shown photographs and sections of them to
very many histologists and pathologists—engaged in general
morphological work, as well as in medical work—and almost
without exception, after looking them over, they say, “ On
casual inspection I should say that they are probably tubular
glands.”
I have observed them in sections of the membrane from man,
dogs, cats, sheep, pigs, about the temporary and permanent
teeth in the young and the old membrane. Like all cellular
elements of the membrane they grow less numerous with age,
but they have been seen in the membrane from a man seventy
years old.
The size, number; persistence and conspicuousness of these
structures make it seem extremely improbable that they are
simply embryonal remains from the epithelial cord or external
or internal tunics of the enamel organ, aB suggested by the work
of Von Brom, quoted by Charles Tomes (Dental Anatomy,
page 107), and as was suggested to me by Dr. Huber, of Ann
Arbor.
It seems to me that these structures must be present for a
purpose, what that may be I can not suggest, and I know noth-
ing that throws any light on the question. In order to work
out the problem to meet the requirements of the histologist four
things must be done :
First.—The origin of the structure must be traced, so as to
determine from what tissue they are derived.
Second.—Their relation to the blood supply must be deter-
mined.
Third.—Their morphology must be determined by making a
complete series of sections, both longitudinal and transverse, to
determine whether they have ducts or not, or other connection
with the gingival epithelium and the complete reconstruction of
them from serial sections. The last task is perhaps impossible,
but it could be done for small area so as to satisfy the demand.
Fourth.—Their condition in diseased states of the mem-
brane must be carefully studied. Until such a program is fol-
lowed out we can but speculate as to what the origin and func-
tion may be, and speculation of this kind does not often aid in
the advancement of scientific knowledge.
				

## Figures and Tables

**Fig. 1. f1:**
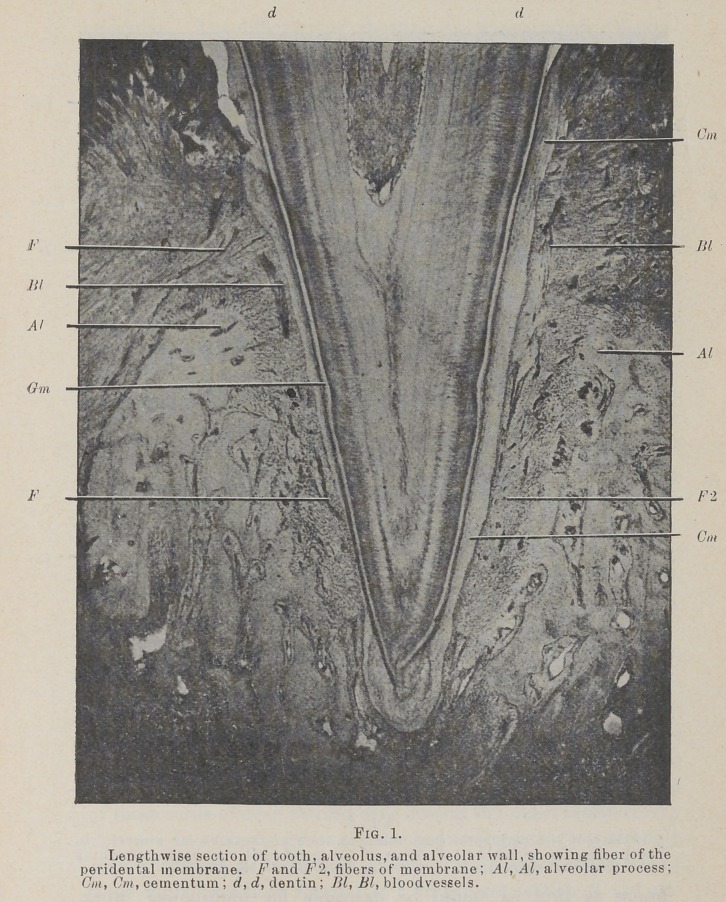


**Fig. 2. f2:**
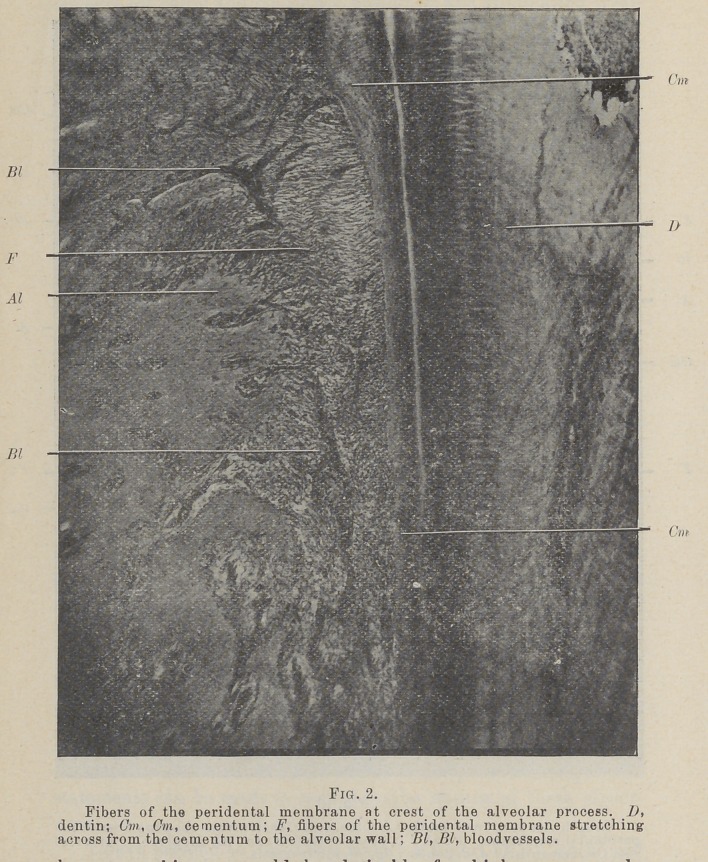


**Fig. 3. f3:**
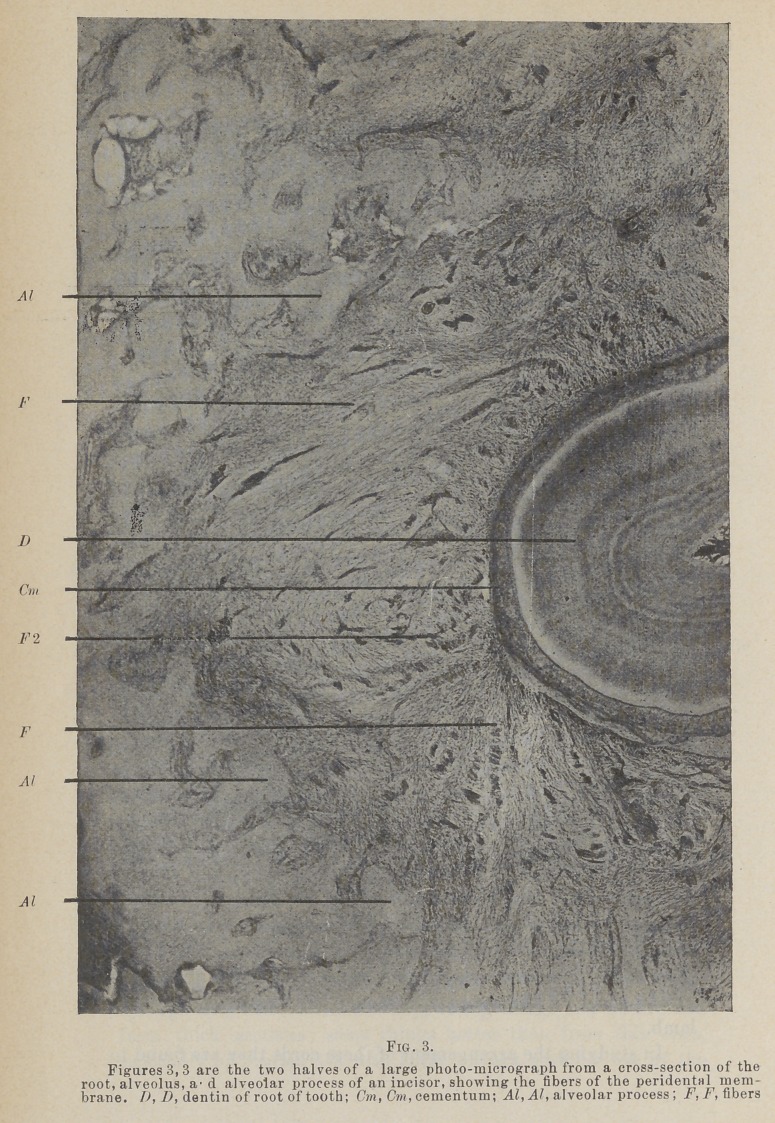


**Fig. 3. f4:**
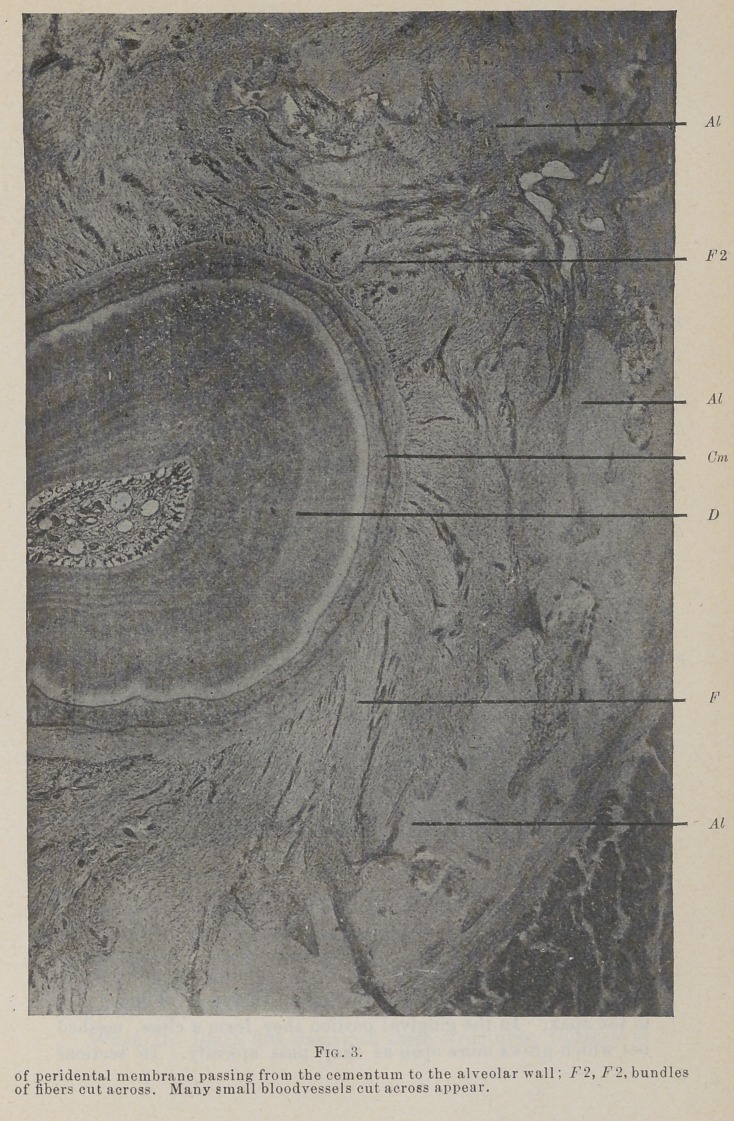


**Fig. 5. f5:**
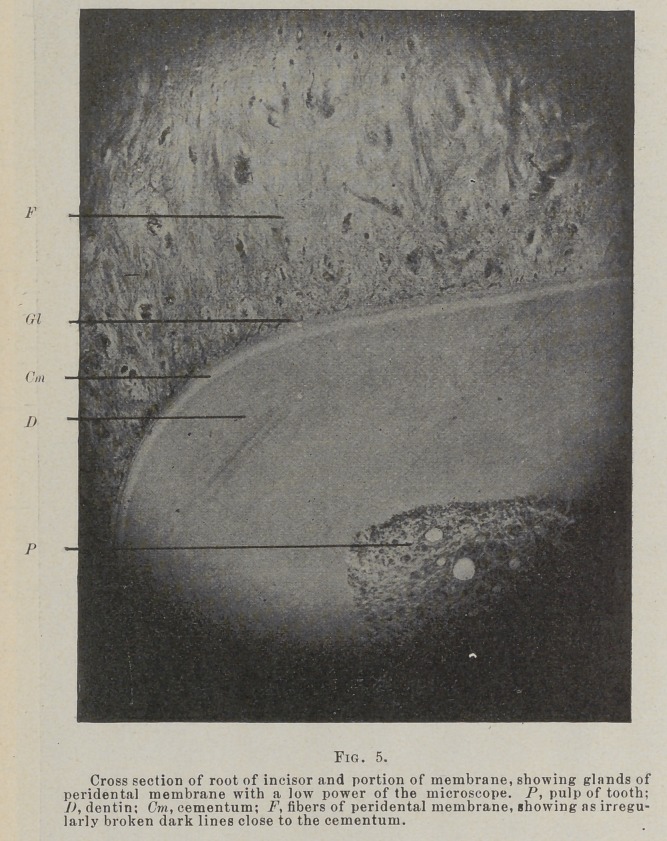


**Fig. 6. f6:**
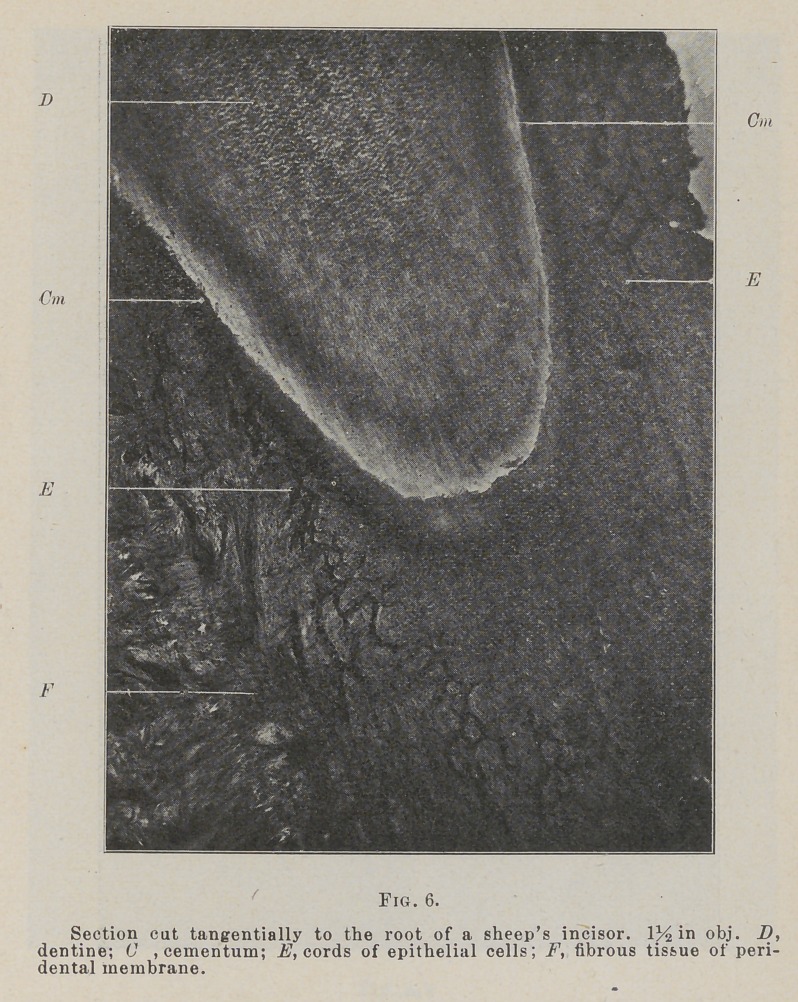


**Fig. 7. f7:**
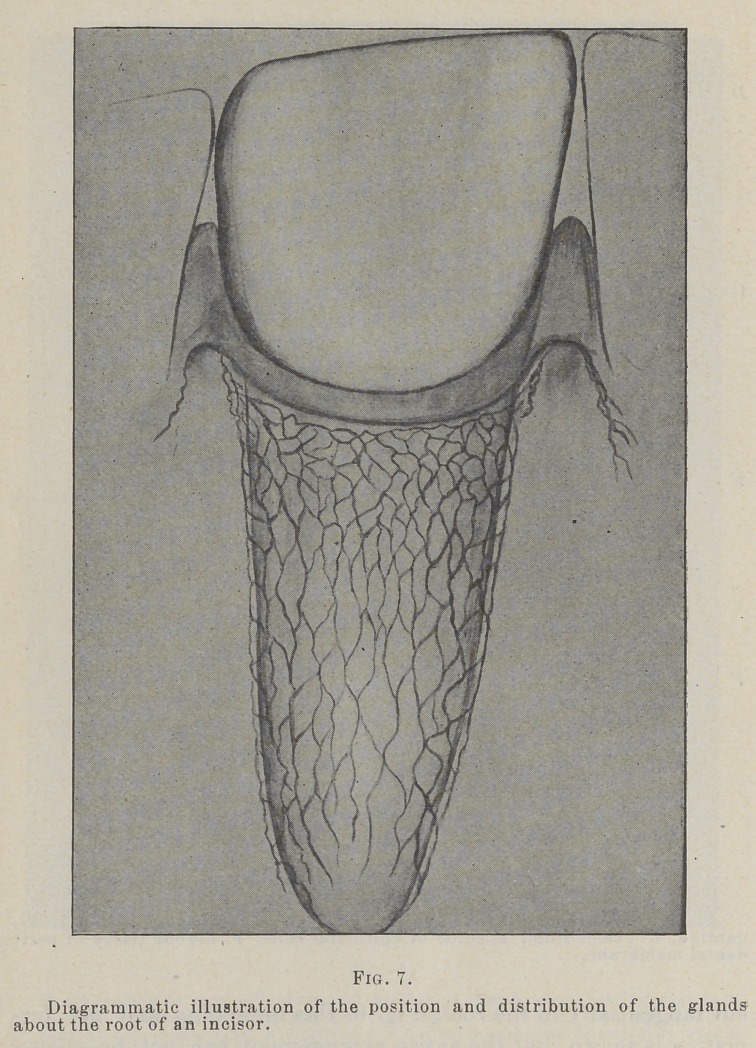


**Fig. 8. f8:**
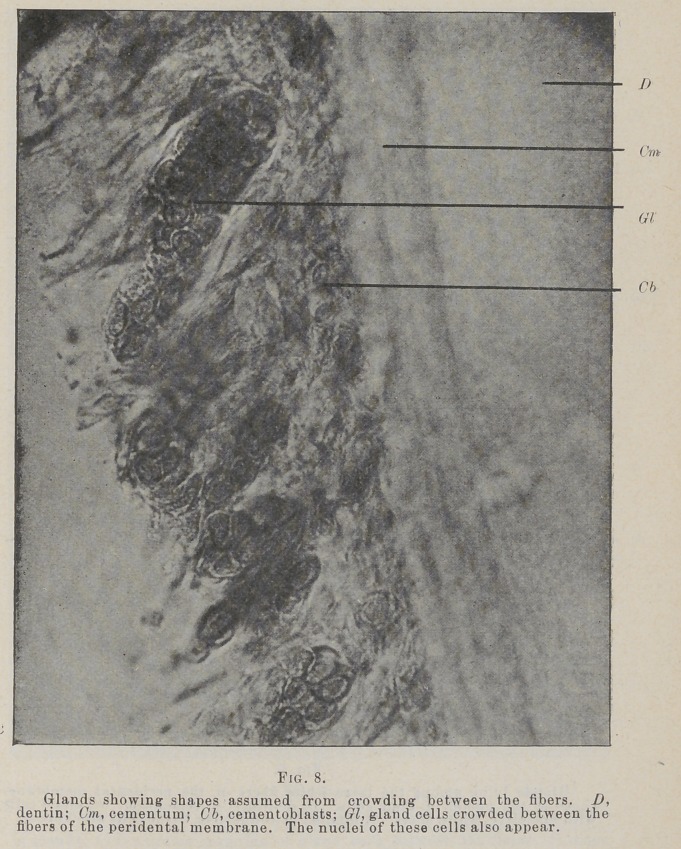


**Fig. 9. f9:**
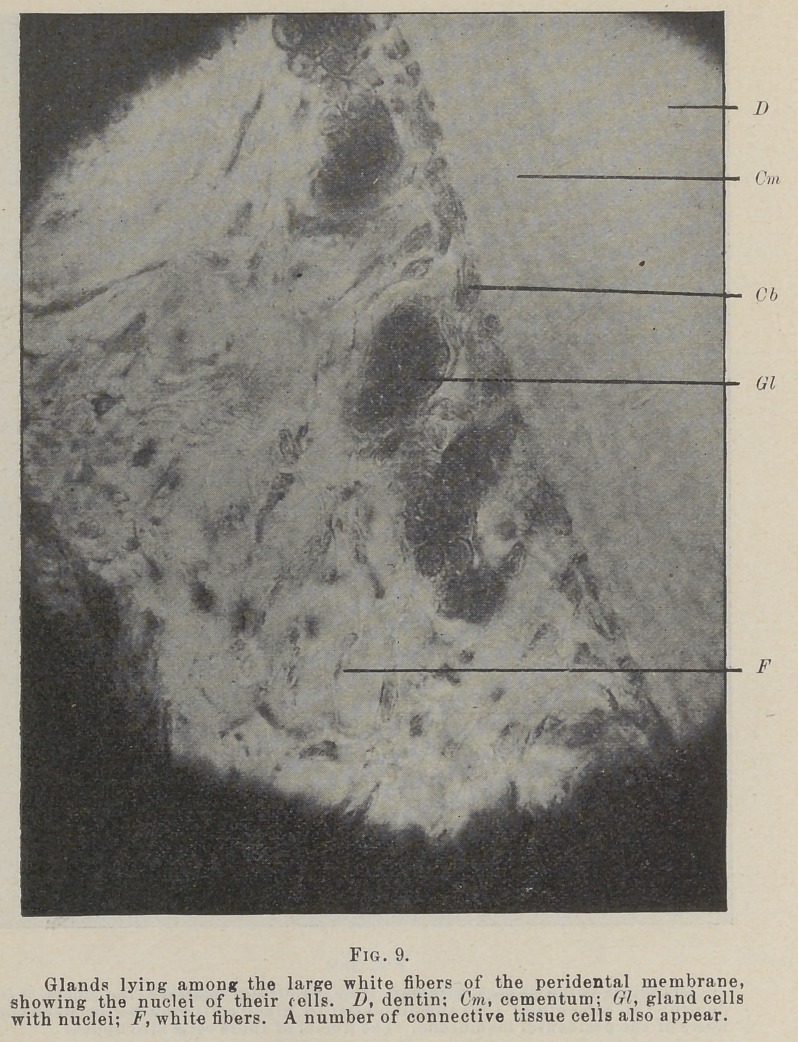


**Fig. 13. f10:**
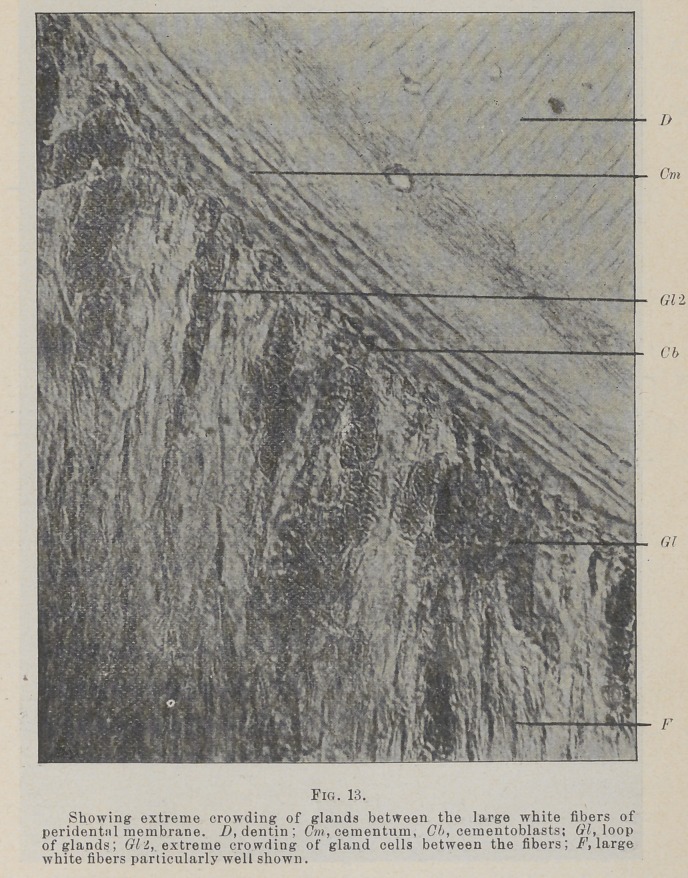


**Fig. 16. f11:**
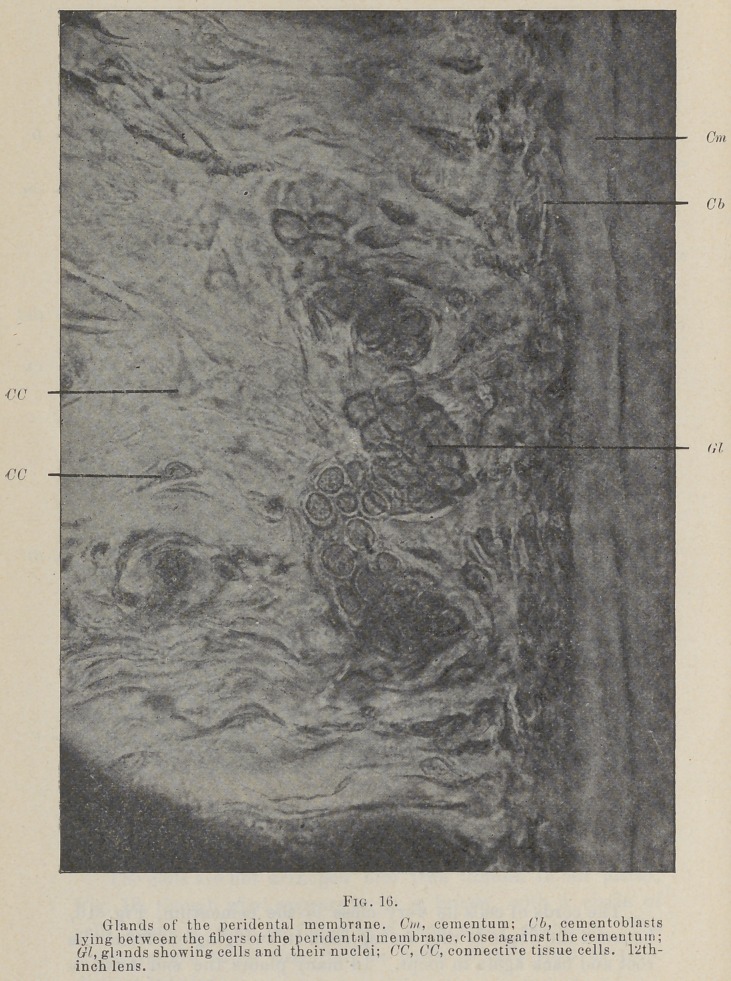


**Fig. 17. f12:**
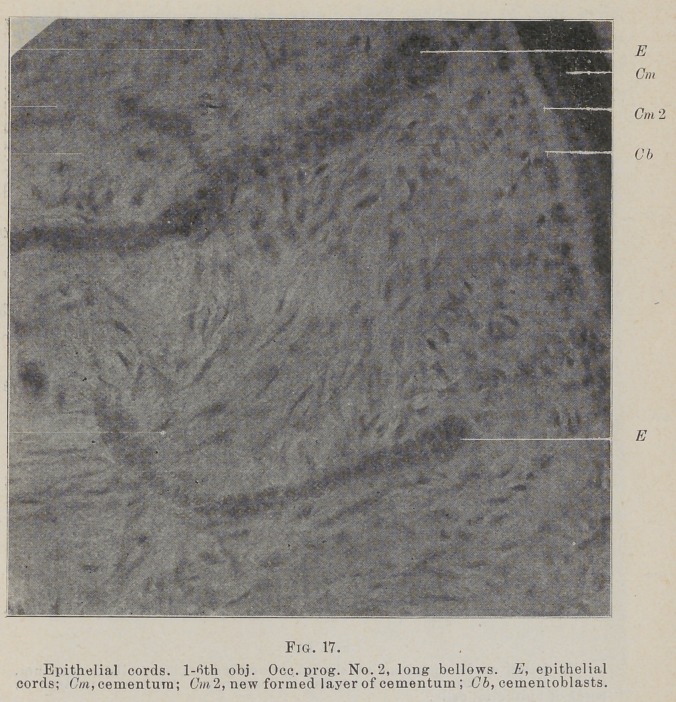


**Fig. 18. f13:**
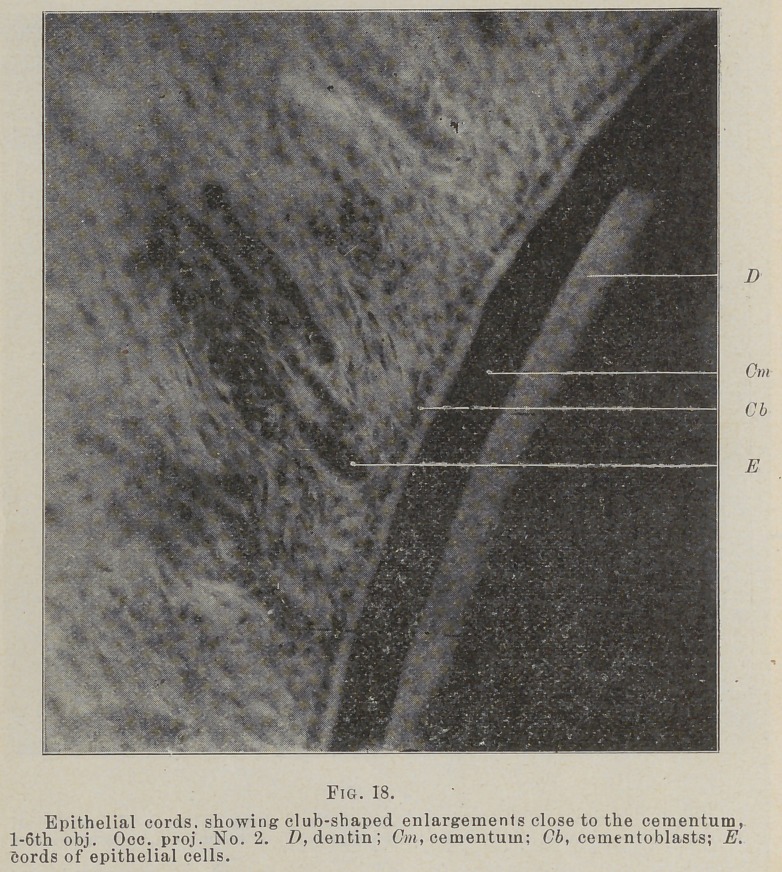


**Fig. 20. f14:**
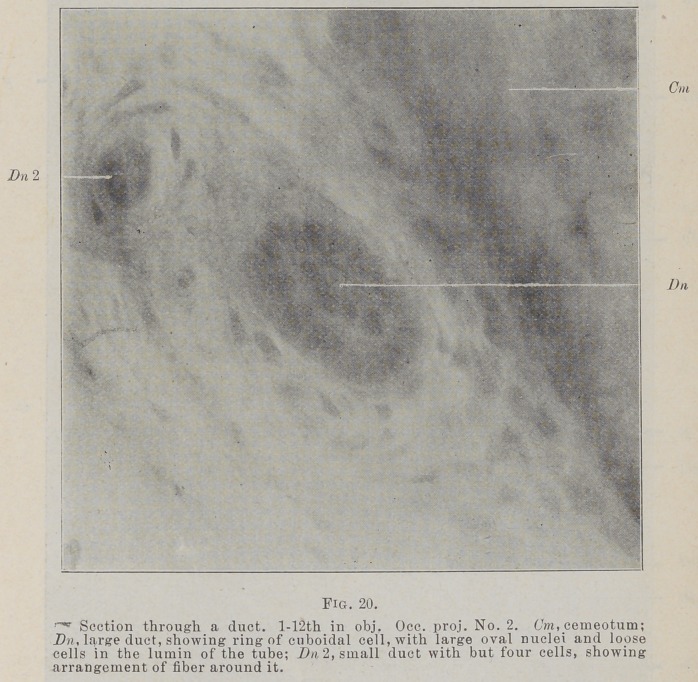


**Fig. 23. f15:**
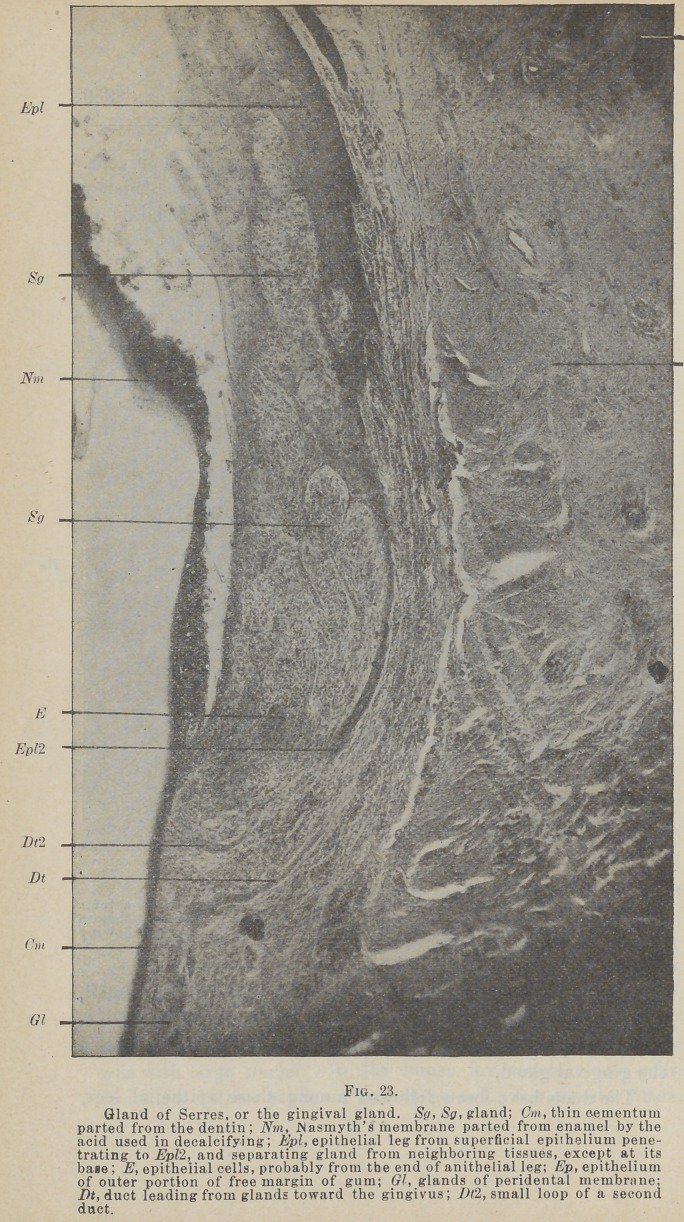


**Fig. 24. f16:**